# Exploring social and public healthcare professionals’ experiences providing pregnancy intentions and contraceptive counseling in the Netherlands

**DOI:** 10.1016/j.xagr.2026.100630

**Published:** 2026-03-18

**Authors:** Mariëlle Cloin, Wendy Jeeninga

**Affiliations:** 1 Tranzo, Tilburg School of Social and Behavioral Sciences (TSB) (Cloin and Jeeninga), Tilburg University, Tilburg, The Netherlands; 2 Regional Public Health Service Hart voor Brabant (Jeeninga), Tilburg, The Netherlands

**Keywords:** contraceptive counselling, motivational interviewing, pregnancy intentions, public health care providers, social care providers, unintended pregnancy prevention, vulnerable populations

## Abstract

**BACKGROUND:**

Unintended pregnancy (UP) is highly prevalent among individuals with multiple social and/or (mental) health challenges. Despite this, pregnancy intentions and contraceptive options are rarely addressed in routine social and public healthcare. To bridge this gap, the Dutch Not Pregnant Now program integrates person-centered counseling on pregnancy intentions and contraceptive options into a variety of social and preventive public health services that people already access, such as social care, mental health and youth care, and public health programs. Since professionals providing these services play a central role in implementation in practice, their experiences and competencies are relevant for understanding feasibility and impact, and to guide further development and implementation of routine counseling on these topics in the Netherlands and beyond.

**OBJECTIVES:**

The aim of this study is to explore social and public healthcare professionals’ self-rated ability to deliver counseling on pregnancy intentions and contraceptive options within the context of the Dutch Not Pregnant Now program. Specifically, we aim to identify both strengths and areas for improvement by examining how professionals’ reported skills, attitudes, and structural barriers relate to their self-perceived counseling capacity, representing core elements and practical requirements for delivering the counseling within their own work setting and during regular client visits, rather than as a separate activity.

**STUDY DESIGN:**

A practice-based, cross-sectional study was conducted using 131 self-reported questionnaires completed by trained professionals providing Not Pregnant Now counseling in social and public healthcare. The survey addressed reported responsibility for discussing pregnancy intentions and contraceptive options during regular care visits, skills in motivational interviewing, competence in contraceptive option counseling, client screening practices, and time constraints. Quantitative data were analyzed using ordinary least squares (OLS) regression to explore factors affecting self-rated counseling ability. Open-ended responses were thematically analyzed to provide additional insights and contextualize the findings within practice.

**RESULTS:**

Social and public healthcare professionals who reported stronger skills in motivational interviewing—particularly in identifying the right moment to initiate the conversation, demonstrating empathy, and applying effective techniques—felt significantly more capable of delivering counseling on pregnancy intentions and contraceptive options during regular care visits. Competence in discussing contraceptive options further enhanced this perceived ability. In contrast, no significant effects were observed for (the lack of) perceived responsibility for providing such counseling, client screening skills, or time constraints. Open-ended responses highlighted the importance of building rapport with clients and creating a nonjudgmental environment to successfully embed counseling into routine care.

**CONCLUSIONS:**

Strengthening and sustaining professionals’ motivational interviewing and contraceptive option counseling skills enhances their confidence and ability to integrate pregnancy intentions and contraceptive option counseling into practice. Embedding these discussions as a normalized component of routine social and public healthcare—services that people already turn to for support—may contribute to more comprehensive, preventive, and person-centered care, supporting unintended pregnancy prevention. The findings highlight the importance of ongoing training, skill development, and normalization of option counseling, not only in the Netherlands but also for other countries seeking to implement this often-overlooked aspect of care within their social and public health systems.

**AJOG Global Reports at a Glance**

**Why was this study conducted?**

Unintended pregnancy prevention is rarely integrated into social and public healthcare services. This study examined how professionals in the Netherlands counsel clients on pregnancy intentions and contraceptive options, focusing on practical ways to strengthen counseling within routine visits.

**Key findings?**

Professionals with stronger motivational interviewing skills felt more capable of counseling during routine visits. Building rapport and a nonjudgmental approach were also key facilitators, while perceived responsibility, screening skills, and time constraints were less relevant.

**What does this add to what is known?**

System-wide unintended pregnancy prevention is limited and fragmented, as is knowledge on what supports professionals in counseling. Examining Dutch professionals’ experiences provides new insights into practical skills and facilitators for integrating such counseling into routine services in the Netherlands and beyond.

## Introduction

Unintended pregnancies (UP), defined as mistimed or unwanted, pose a major burden on individuals, families, healthcare systems, and society.^1^ In Western countries, ∼40% of pregnancies are unintended.^2^ Risk factors include young age, low socioeconomic status, and adverse conditions such as poverty, homelessness, substance use, psychiatric vulnerability, intellectual disabilities, and (sexual) violence.^3^, ^4^, ^5^, ^6^ Explanations include stress from precarious living conditions, inconsistent contraceptive use, misjudgment of pregnancy risks, limited perceived reproductive control, and financial barriers.^1^^,^^7^

Several studies identify the lack of general UP prevention as a system failure, particularly for high-risk populations.^8^, ^9^, ^10^ Few initiatives target comprehensive implementation across (social) care systems, despite evidence suggesting that such an approach is necessary.^3^^,^^11^, ^12^, ^13^ For example, the "One Key Question" (OKQ) screening tool from the United States supports healthcare providers to proactively address reproductive health by asking women of reproductive age, "Would you like to become pregnant in the next year?" to identify intentions and guide counseling to contraceptive or preconception care.^14^^,^^15^ Although its potential application is broader, in practice it is mostly used in clinical settings.

Targeting the broader group of social and public healthcare clients, the “Not Pregnant Now” program in The Netherlands (in Dutch Nu Niet Zwanger, abbreviated NNZ^16^) involves person-centered counseling by social care and public health professionals from a diverse range of services, including mental health, substance use, poverty/homelessness, domestic violence/child abuse, intellectual disability care, sexual healthcare, and preventive youth healthcare. Launched as a pilot in Tilburg (2014), NNZ became part of the national “Solid Start” program in 2018 and by 2023 was adopted by 66% of municipalities. As of 2025, it is embedded in the Supplementary Health and Social Care Agreement (AZWA) for nationwide implementation. Core to the program is that social and public healthcare providers proactively discuss pregnancy intentions and contraceptive options during regular visits using person-centered counseling and motivational interviewing techniques. This implies asking open-ended questions, tailoring conversations to client responses, being nondirective, not judgmental, and having interpersonal relationships and trust as key elements, opposed to more directive forms of communication.^1^^,^^4^^,^^9^^,^^17^^,^^18^ It also includes follow-up services, such as referrals to preconception care, aligned GP/obstetric services, (same day) contraceptive services, and financial assistance for clients facing challenges. Although the program primarily serves women in practice, its framework is gender-inclusive, supporting male clients and partners when appropriate.

Despite the program’s potential to support broad UP prevention, little is known about professionals’ experiences in practice, and the literature also provides fragmented insights. Some studies from reproductive healthcare address preconception or contraceptive counseling for specific groups such as teenage mothers or people with health problems,^6^^,^^19^^,^^20^ while others focus on specific methods like long-acting reversible contraception.^12^ Outside reproductive healthcare, research shows that providers often lack knowledge, training, or counseling skills,^19^^,^^21^, ^22^, ^23^, ^24^, ^25^ are uncertain whom to counsel,^19^^,^^21^^,^^25^^,^^26^ or view it as outside their professional scope.^19^^,^^21^^,^^22^^,^^23^^,^^26^^,^^27^ Across clinical and nonclinical settings, time constraints and competing priorities are also reported.^15^^,^^19^^,^^21^^,^^22^^,^^23^^,^^26^ In sum, there is limited and fragmented understanding of experiences with UP prevention counseling, particularly among social care and public healthcare providers.^1^^,^^11^^,^^22^^,^^25^ Therefore, this study explores social care and public healthcare professionals' self-rated ability to counsel clients on pregnancy intentions and contraceptive options in the context of the Dutch NNZ program. Beyond the specific program, this knowledge can inform existing and new (international) settings that want to integrate person-centered counseling in routine social and/or (public) health care.^22^^,^^28^

## Materials and methods

### Survey

We use data from an online questionnaire completed by Dutch social and public healthcare professionals in the pilot region (Tilburg/Midden-Brabant) in September and October 2019. The questionnaire was based on existing literature, previous studies, and the core elements of the program as described in NNZ protocols. For instance, several quantitative studies informed our survey items on professionals’ perspectives on their own counseling practices, perceived importance and responsibility of such counseling, training needs, knowledge of contraceptive options, and time constraints, while qualitative studies informed questions on identifying clients to counsel, motivational interviewing, and fostering nonjudgmental client-professional relationships.^21^^,^^25^^,^^27^, ^28^, ^29^ The questionnaire next was reviewed and tested by experts, including birth/healthcare professionals (a gynecologist, a midwife/coordinator of a regional Birth Care Consortium), a local and the national NNZ coordinator, senior researchers, and 3 women with prior experience of NNZ counseling, ensuring relevance and clarity of the questions relevant for real-world implementation. For instance, questions were asked about knowledge and skills in motivational interviewing, understanding of contraceptive options, whether they felt they were reaching the appropriate clients, and whether they had sufficient time and support to carry out the counseling.

A convenience sample method was used. Within each organization, decisions on which staff members provided counseling were made internally (eg, based on caseload or role responsibilities). As no formal records of the total staff involved were available, the questionnaire was distributed via 1 or 2 designated NNZ professionals in each of the 19 organizations formally committed to implementing the program (18 social service organizations and 1 regional public health service). These professionals invited colleagues by email, explained the study’s purpose, voluntary participation, and right to withdraw, and provided a link to the online informed consent form and questionnaire. A potential risk is selection bias, whereby more engaged or motivated professionals may be more likely to participate, potentially leading to more positive counseling experiences. Nevertheless, for exploratory purposes, we were able to ensure variation in professional experience with the program, and involvement across social and public healthcare services. (see Table 1)."

**Table 1:** Descriptive summary statistics of the variables in the study (n=131).

	range (min-max)	mean (SD) or % (n)
Perceived ability to have an open conversation on pregnancy intentions and contraceptive options with clients	0–100	73.94 (20.92)
Perceived responsibility for providing the program		
I feel that the organization I work for is responsible for identifying and supporting the target group of Not Pregnant Now	0–100	81.68 (19.57)
I feel personally responsible for identifying and supporting the target group of Not Pregnant Now	0–100	77.29 (21.91)
Identification of clients as eligible for the program		
I am sufficiently informed on how to identify clients eligible for the program	0–100	76.64 (24.32)
I am successful in identifying clients eligible for the program	0–100	76.03 (21.97)
*Program knowledge & skills*		
Knowledge and skills: motivational interviewing		
I am sufficiently informed on how to have an open conversation on pregnancy intentions and contraceptive options with clients	0–100	74.81 (24.25)
I am sufficiently informed on the interview techniques to use in conversations on pregnancy intentions and contraceptive options with clients	0–100	67.79 (28.35)
I am successful in empathizing with the living conditions of clients that are eligible for the program	0–100	70.38 (24.97)
I am successful in using appropriate interviewing techniques in conversations on pregnancy intentions and contraceptive options with clients	0–100	70.23 (24.10)
I am successful in supporting clients in their voluntary contraceptive options decision-making process	0–100	61.83 (24.99)
Knowledge and skills: contraceptive options		
I am sufficiently informed on different contraceptive options	0–100	61.07 (32.19)
I am successful in informing clients on different contraceptive options	0–100	63.36 (28.52)
I am sufficiently informed on the pathways to realize contraception for clients in the context of Not Pregnant Now	0–100	58.93 (31.01)
I am sufficiently informed on the financial resources available within the program for contraceptive methods.	0–100	52.37 (33.14)
Time and implementation in practice		
I have enough time to implement Not Pregnant Now in my daily work	0–100	44.58 (30.03)
I feel Not Pregnant Now should be a structural part of my daily work	0–100	80.76 (20.67)
*Control variables*		
Dedicated professional (ref.=yes)	0–1	34.4 (45)
Organization (ref.=RHS)	0–1	45.0 (59)
Length of experience (months)	0–50	12.1 (10.22)
Number of clients supported	1–5	3.07 (1.38)

To safeguard anonymity, no demographic data were collected. Yet months of experience and number of clients counseled (see Control variables) are indicative of seniority with the program. For gender, it is worth noting that most professionals in public health and social work are women according to Statistics Netherlands (76%–93%). Ethical approval was obtained from the Ethics Review Board of Tilburg University [TSB_RP681]. The final sample consisted of 131 care providers, 72 social care providers, and 59 public healthcare providers. Respondents originated from public health (45%, from the Regional Public Health Service in the area), with professionals like youth healthcare nurses, doctors, and sexual health professionals. Social workers came from social services addressing intellectual disabilities (28%), psychiatric vulnerability (10%), youth care and domestic violence (9%), and other organizations focusing on poverty, homelessness, substance use, and probation (8%).

## Measures

### Professionals self-rated ability in counseling

Professionals’ ability to counsel was operationalized, following expert guidance and the NNZ protocols, as their self-rated ability to have an open conversation with clients about pregnancy intentions and contraceptive options. This was assessed with 3 similarly matched questions: “I am successful in having an open conversation with clients about pregnancy intentions”, ”I am successful in having an open conversation with clients about sexuality“ and “I am successful in having an open conversation with clients about contraceptive options.” Answers were rated on a 5-point Likert scale with 1=strongly disagree, 2=disagree, 3=neutral, 4=agree, and 5=strongly agree. A factor analysis (PCA) identified 1 latent factor underlying the data, with an eigenvalue of 2.302 and 76,73% variance explained. KMO was .73 and Bartlett’s test of sphericity was significant (χ2 (3)=168.88, *P*<.001). The internal consistency was good (Cronbach’s α=0.846). The individual scores of the professionals are standardized, with a mean of 0 and a standard deviation of 1, and transformed with min-max normalization, rescaling the individual factor scores within the range 0 to 100. This construct "professionals" self-rated ability to counsel’ reflects the sampled subgroup rather than the entire professional population working within the program, although variation in scale scores is present (see Table 1).

### Program elements and practice

Fifteen key components of the program were examined in practice to explore how they relate to social and public healthcare professionals’ experiences in providing counseling (see Table 1). These are categorized into "self and organizational responsibility for providing the program" (2 items), "knowledge and skills for client identification" (2 items), and "program information, knowledge, and skills," including 5 items on motivational interviewing and counseling techniques, and 4 items on knowledge and skills about contraceptive options and contraception realization. Finally, time constraints (1 item) and program integration into daily work were considered (1 item). All 15 items were assessed on a 5-point Likert scale ranging from 1 ("totally disagree") to 5 ("totally agree"). All items were rescaled to a 0 to 100 range. This standardization reflects respondents’ relative positions on the response continuum, with higher scores indicating stronger endorsement, comparable across all items, and enables direct comparison of program elements in relation to professionals’ self-rated counseling ability.

### Control variables

Control variables included length of experience with the counseling and number of clients supported, as these factors may impact professionals' perceived counseling ability.^29^^,^^30^ Indicated months of experience (open question) varied between 0–50. The number of clients supported was assessed by asking “With how many clients did you have an open conversation about pregnancy intentions and contraceptive options so far?”, where: 1=none, 2=1 to 5 clients, 3=6 to 10 clients, 4=11 to 15 clients, 5=> 15 clients. Also, we control for the clustering of respondents working at public health (ie, RHS=1, ref. cat) versus social services.

### Open ended text responses

We used text responses from the survey asking respondents to indicate what they value, find most difficult, and need most in the program to help clarify and further interpret the quantitative results. The text responses tended to be brief, containing a single reaction closely aligned to the themes of the survey. With little risks for misinterpretation or subjectivity, the presented responses were selected by the second author and verified by the first author.

### Statistical methods

Descriptive statistics summarize the mean scores and Ordinary Least Squares (OLS) regression is used using SPSS Statistics 27. OLS regression was used because the dependent variables derived from the PCA is a continuous variable (ranging from 0–100), making OLS an appropriate and commonly used method.”

## Results

### Descriptive results

Table 1 displays professionals' self-rated ability to counsel, with a mean score of 73.94 (SD 20.92) on a scale from 1 to 100. Professionals feel largely responsible for the counseling, both individually (mean 77.29) and at the organizational level (mean 81.68), meaning they see their organization as co-responsible for UP prevention and the related counseling provided during regular care visits. They demonstrate moderate to high knowledge and skills in identifying eligible clients for the counseling in their caseload (76.64 and 76.03) and motivational interviewing and counseling techniques (61.83–74.81). Mean scores are lower for knowledge on contraceptive options (61.07), informing clients on options (63.36), pathways to contraception (58.93), and financial resources (52.37). The mean score for having the time to counsel is low (44.58), but high for the necessity to integrate the counseling into daily work (80.76).

### Ordinary least squares (OLS) regression analysis and text responses

The OLS model explained a significant amount of variance in professionals self-rated ability to counsel their clients (F(2, 17)=4.63, *P*<.05, R2=.59, R2Adjusted=.28) (Table 2). Neither the 2 perceived responsibility items nor the 2 client identification items significantly influenced professionals self-perceived counseling ability. Explanations could be that many professionals indeed endorse the program and know who to counsel because they already have an established relationships with clients through regular care visits. This is highlighted in some of the open-ended text responses:

**Table 2:** Ordinary least squares regression model on potential barriers and facilitators for professionals self-rated counseling ability (n=131).

	B	SE	β	t	*P*-value
Perceived responsibility for program delivery					
I feel that the organization I work for is responsible for identifying/ signaling and providing support to the target group of Not Pregnant Now	–0.124	0.286	–0.031	–0.433	.666
I feel personally responsible for identifying/ signaling and supporting the target group of Not Pregnant Now	–0.580	0.296	–0.177	–1.963	.052
Identification of clients as eligible for the program					
I am sufficiently informed on how to identify clients eligible for the program	–0.149	0.236	–0.058	–0.631	.529
I am successful in identifying clients eligible for the program	0.052	0.236	0.018	0.219	.827
*Program knowledge & skills*					
Knowledge & skills for motivational interviewing / counseling					
I am sufficiently informed on how to have an open conversation on pregnancy intentions and contraceptive options with clients	0.612	0.238	0.236	2.572	.011⁎⁎
I am sufficiently informed on the interview techniques to use in conversations on pregnancy intentions and contraceptive options with clients	–0.075	0.223	–0.034	–0.337	.737
I am successful in empathizing with the living conditions of clients that are eligible for the program	0.574	0.187	0.228	3.064	.003⁎⁎
I am successful in using appropriate interviewing techniques in conversations on pregnancy intentions and contraceptive options with clients	0.608	0.237	0.233	2.566	.012⁎⁎
I am successful in supporting clients in their voluntary contraceptive options decision–making process	–0.033	0.205	–0.013	–0.161	.872
Knowledge & skills on contraceptive options					
I am sufficiently informed on different contraceptive options	–0.259	0.154	–0.133	–1.683	.095*
I am successful in informing clients on different contraceptive options	0.668	0.200	0.303	3.342	.001⁎⁎
I am sufficiently informed on the pathways to realize contraception for clients in the context of Not Pregnant Now	0.195	0.166	0.097	1.179	.241
I am sufficiently informed on the financial resources available within the program to receive reimbursement for contraceptive methods.	–0.272	0.162	–0.144	–1.681	.096*
Time and implementation in practice					
I have enough time to implement Not Pregnant Now in my daily work	–0.004	0.130	–0.002	–0.027	.978
I feel Not Pregnant Now should be a structural part of my daily work	0.816	0.259	0.269	3.147	.002⁎⁎
Control variables					
Dedicated professional (ref.=yes)	–0.403	0.534	–0.061	–0.755	.452
Organization (ref.=RHS)	–1.551	0.410	–0.247	–3.783	.000⁎⁎⁎
Length of experience (months)	–0.028	0.024	–0.094	–1.200	.233
Number of clients supported	0.398	0.165	0.176	2.413	.017⁎⁎

⁎ P<.1

⁎⁎ P<.05

⁎⁎⁎ P<.001.

*“Thanks to the program, things have changed, and we are now more aware of unintended pregnancies or a desire to have children. We feel more responsible for our preventive role.” - Public health professional*

Three of the 5 motivational interviewing and counseling knowledge and skills items yielded positive effects. Feeling sufficiently informed on how to start the conversation (Beta =.236, t(2.57) *P*=.011), being able to relate and be empathetic towards clients (Beta=.228, t(3.07) *P*=.003), and being able to use the appropriate interviewing techniques (Beta=.233, t(2.57) *P*=.012) positively influences professionals self-rated ability to counsel clients:

“*Taking time is very important and trust is the key word. Building a relationship, not interfering and creating awareness are important. And letting them make their own choices*”. *- Social professional*

“*Being genuinely interested in their story gives a lot of space for the other person to think and make choices. Sometimes you also see relief; that it is allowed / possible to talk about it.” - Social professional*

In addition, many professionals expressed a need for (continuing) professional training, in depth case reflection and supervision to keep up or fill gaps in knowledge and skills:

*“Sharing information and experiences, learning from each other. I think that will help me get better at it.” - Social professional*

*“I need consultation with other professionals; how do they start an open conversation? We can learn from each other.” - Social professional*

Moreover, professionals' self-rated ability in option counseling was significantly influenced by their skills related to counseling on contraceptive options (Beta=.303, t(3.34), *P*=.001). However, knowledge on different contraceptive options, financial resources for contraception, or how to realize contraception, did not.

*“[What I value is] being able to help the client to find appropriate contraception that the client is satisfied with.” - Social professional*

Time constraints had no significant effect, which might indicate that other issues underly real- or perceived-time constraints. For example, as these quotes indicate, professionals do identify time as being relevant, yet often in conjunction with issues related to discomfort, (organizational) priorities or motivation:

*“It's partly time and partly a matter of “how do I do it." Of course, if you feel that way, you can very easily think "Well, then I won't do it." - Public health professional*

Furthermore, professionals in favor of integrating the program into their daily work reported a higher self-rated counseling ability (Beta=.269, t(3.15), *P*=.002). This finding is consistent with feedback from some professionals:

*“I think by staying alert and being reminded of it helps to eventually integrate it well into daily activities, it needs time to sink in.” - Social professional*

For the control variables, the results showed that social professionals felt less successful in counseling (having open conversations) compared to public health professionals. More clients supported was positively associated with a higher self-perceived counseling ability, but length of experience in months and being affiliated as a dedicated professional to the program did not have significant effects.

## Discussion

### Principal findings

This study examined social and public healthcare professionals’ experiences with person-centered counseling on pregnancy intentions and contraceptive options during regular care visits. Skills in motivational interviewing, empathy, contraceptive knowledge, and integrating counseling into daily work shaped their self-rated ability. Public health professionals felt more confident than social care providers, highlighting the need for ongoing training and support.

## Rasults

The findings align with previous research on the importance of counseling skills and expertise.^21^^,^^22^^,^^29^ Unlike earlier studies, lack of time was not related to self-rated ability, possibly masking issues such as limited training, workload, or organizational support.^15^^,^^22^ While perceived responsibility and the ability to signal clients for counseling were rated as high, these factors did not influence self-rated counseling ability in our study. This contrasts with previous research, where such factors were found to be key determinants.^22^^,^^26^ This could be because professionals in public health and social services already have established relationships with their clients, their support needs, and are familiar with their backgrounds. Our findings highlight the importance and added value of embedding counseling structurally within such routine care visits, rather than standalone initiatives (specific target groups, providers or setting) or short-term projects/time-limited training programs.^19^^,^^23^ Embedding counseling structurally within routine preventive care may provide a particular favorable context for counseling, also as for instance, in primary care and obstetrics–gynecology practice, providers have limited contact with individuals prior to an unintended pregnancy, a finding also reported in earlier studies.^1^^,^^8^

## Clinical implications

We found that public health professionals feel better able to provide UP prevention counseling compared to social care providers. This may be understandable, given public health professionals’ training and focus on topics such as sexual health, family planning, and parenting, as opposed to social care providers, for whom these topics often fall outside their scope of practice. This suggests that public health settings are a promising context for delivering such counseling, while implementation in social care may require more effort, and underscores the importance of addressing the needs of professionals to enhance UP prevention counseling in various settings. This also includes the collaboration with birth care professionals in signaling, referral, and, where appropriate, providing contraceptive services.

## Research implications

Future research is needed using larger, more representative (probability) samples to confirm and deepen our explorative findings, to better understand the factors underlying professional experience in counseling, and to inform strategies to further improve, implement, and sustain program delivery. This should also include more professionals from primary care and obstetrics-gynecology, and consider factors not captured here, such as professionals’ personal beliefs about contraception, ethical dilemmas, comfort in discussing reproductive topics, and language barriers.^24^^,^^26^ Longitudinal studies could also explore how counseling experiences evolve over time.

## Strengths and limitations

A strength of this study is its focus on professionals across social and public healthcare and the practical assessment of counseling experiences. Limitations include the use of a convenience sample, which may bias participation toward those more involved or with stronger opinions, and the cross-sectional design capturing experiences at a single point in time.

## Conclusions

This study provides exploratory insights from a diverse range of social and public healthcare professionals delivering UP prevention counseling during routine care visits. It highlights the importance of practical skills, organizational support, and context-specific implementation strategies to enhance the reach and effectiveness of person-centered counseling on pregnancy intentions and contraceptive options.

## Patient consent statement

This study was based on anonymized survey data from professionals and did not involve the use of individual patient data. However, clients who contributed to the development of the survey questionnaire provided informed consent.

## Declaration of generative AI and AI- assisted technology in the writing process

The author(s) verify and take full responsibility for the use of generative AI in the preparation of this manuscript. ChatGPT was used in order to improve flow and conciseness of the content. After using this tool, the authors reviewed and edited the content as needed and take full responsibility for the content of the publication.

## CRediT authorship contribution statement

**Mariëlle Cloin:** Writing – original draft, Methodology, Funding acquisition, Formal analysis, Data curation, Conceptualization. **Wendy Jeeninga:** Writing – review & editing, Validation, Funding acquisition.

## Declaration of competing interest

The authors reports no conflict of interest.
